# Impact of Resistance Exercise and Nitrate Supplementation on Muscle Function and Clinical Outcomes After Knee Osteoarthritis Surgery in Middle-Aged Women with Sarcopenia: A Randomized, Double-Blind, Placebo-Controlled Clinical Trial

**DOI:** 10.3390/jcm14020615

**Published:** 2025-01-18

**Authors:** Han-Soo Park, Jin-Ho Yoon, Jae-Keun Oh

**Affiliations:** Sports Medicine Laboratory, Korea National Sport University, 1239 Yangjae-daero, Songpa-gu, Seoul 05541, Republic of Korea; hansoo85@hanmail.net (H.-S.P.); tkd97@knsu.ac.kr (J.-H.Y.)

**Keywords:** nitrate, nitric oxide, beet juice, sarcopenia, sarcopenic osteoarthritis, resistance exercise

## Abstract

**Background/Objectives:** Sarcopenia, characterized by reduced muscle mass and strength, is associated with osteoarthritis (OA), particularly in middle-aged women, and may worsen postoperatively. Resistance exercise (RE) can resolve sarcopenia; however, recovery is often suboptimal. Nitrate (NO_3_^−^) supplementation may enhance muscle recovery and complement RE. We investigated whether NO_3_^−^ supplementation combined with RE improves thigh muscle mass and strength in middle-aged women during postoperative rehabilitation. **Methods:** We conducted a prospective randomized placebo-controlled double-blind study including 36 middle-aged women with sarcopenia and cartilage defects undergoing mesenchymal stem cell implantation. Participants were assigned to RE with NO_3_^−^ supplementation (NG, *n* = 18) or with placebo (PG, *n* = 18) groups. Both groups underwent 12 weeks of supervised RE. The primary outcomes were thigh muscle cross-sectional area (CSA) and knee strength, whereas functional and clinical measures, including the Short Physical Performance Battery (SPPB), skeletal muscle index (SMI), International Knee Documentation Committee (IKDC), and Western Ontario and McMaster Universities Osteoarthritis Index (WOMAC) scores, were secondary outcomes. **Results:** Thigh muscle CSA decreased in the PG but was maintained in the NG. Knee extension strength improved significantly in the NG compared with that in the PG at 6 and 12 weeks. Knee flexion strength also improved rapidly in the NG, with a significant increase at 6 weeks. SPPB and IKDC scores improved significantly in the NG. However, similar improvements were observed for WOMAC scores in both groups. **Conclusions:** NO_3_^−^ supplementation combined with RE effectively prevented muscle atrophy and enhanced muscle strength in our study participants, indicating potential for improving postoperative recovery.

## 1. Introduction

Sarcopenia, which is a muscle disease following the International Classification of Diseases criteria, is characterized by a progressive decline in skeletal muscle mass and strength. It has an increased prevalence in older populations; however, muscle mass decline begins after 40 years of age, significantly impacting the quality of life, healthcare needs, and morbidity in middle-aged and older adults [1].

Notably, sarcopenia is strongly associated with osteoarthritis (OA), particularly in middle-aged women. In addition, low thigh muscle mass has more influence on the severity of knee OA than obesity [2,3].

Surgical treatments, including mesenchymal stem cell (MSC) implantation, are often needed for advanced OA. This regenerative approach is particularly suitable for younger and active patients and is more beneficial than total knee replacement because revision risks are minimized, and the joint integrity is preserved [4].

However, sarcopenia can negatively impact surgical outcomes. It exacerbates postoperative complications and impedes recovery [5,6]. Additionally, postoperative catabolism worsens with the use of pneumatic tourniquets, including prolonged non-weight-bearing protocols; accelerates muscle mass loss; and weakens muscle strength due to inflammation, edema, and arthrogenic muscle inhibition [7,8]. Hence, appropriate strategies to prevent muscle atrophy and preserve strength in these middle-aged women are important.

Furthermore, resistance exercise (RE) is an established strategy that counteracts sarcopenia by enhancing protein synthesis and improving physical functions, such as gait speed and balance [9]. Nonetheless, postoperative recovery often remains suboptimal, with muscle deficits persisting for several years, possibly hindering the restoration of optimal functional capacity and overall quality of life [10,11].

Emerging evidence suggests that nitrate (NO_3_^−^) supplementation may enhance muscle recovery by improving muscle contraction efficiency, nutrient delivery, and calcium metabolism. Additionally, NO_3_^−^ reduces muscle fatigue through its effects on creatine phosphate efficiency [12,13,14]. However, the role of NO_3_^−^ supplementation in postoperative rehabilitation, particularly when combined with RE, remains unclear despite these promising findings. The challenges posed by sarcopenia on recovery and functional outcomes following OA surgery are significant. Hence, there is a critical need to investigate whether NO_3_^−^ supplementation can provide additive or synergistic benefits when combined with RE. Therefore, we hypothesized that combining NO_3_^−^ supplementation with RE during postoperative rehabilitation would be more effective than placebo supplementation in improving muscle function and clinical outcomes in middle-aged women with sarcopenia.

## 2. Materials and Methods

### 2.1. Study Design and Blinding

We conducted a prospective, randomized, placebo-controlled, double-blind trial and performed all procedures at the Sports Medical Center of an Orthopedic Hospital (JS Hospital, Seoul, Republic of Korea). Patients underwent MSC implantation to treat OA, and all interventions were performed by therapists who were not included in the study.

**Figure 1:**
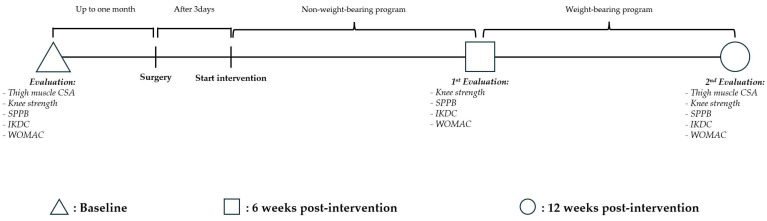
Experimental design. CSA: cross-sectional area; SPPB: Short Physical Performance Battery; IKDC: International Knee Documentation Committee; WOMAC: Western Ontario and McMaster Universities Osteoarthritis Index.

Similarly, groups were randomly assigned by a statistician who was not involved in this study. Participants were assigned to the RE with NO_3_^−^ supplementation group (NG) and the RE with placebo supplementation group (PG) using the Research Randomizer program (https://www.randomizer.org/, accessed on 20 October 2023). Neither the researcher nor the participants were aware of their group allocation because of the study’s blinding procedure. Assessments were conducted at baseline and at 6 and 12 weeks postintervention (Figure 1).

**Figure 2:**
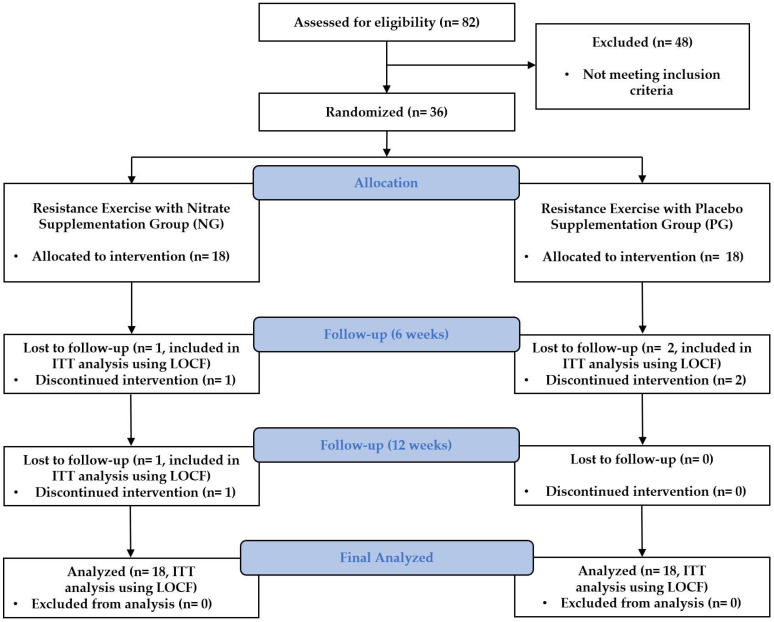
CONSORT flow diagram. LOCF: last observation carried forward; ITT: intent-to-treat.

All procedures in this study followed the principles of the Declaration of Helsinki and were approved by the Institutional Review Board of the Korea National Sport University (20230621-072). The Clinical Research Information Service approved the clinical trial registration before the first participant was enrolled (KCT0008830). The Consolidated Standards of Reporting Trials flow diagram for this study is shown in Figure 2.

### 2.2. Participants

We included women aged 45–65 years with sarcopenia diagnosed with grade IV cartilage defect in the medial or lateral femoral condyle based on the International Cartilage Repair Society (ICRS) guidelines. Participants were scheduled for MSC implantation within 1 month. Furthermore, we defined sarcopenia as a skeletal muscle index (SMI) of <5.7 kg/m^2^, handgrip strength of <18 kg, or a Short Physical Performance Battery (SPPB) score of <9 points.

The exclusion criteria were as follows: severe OA (Kellgren–Lawrence stage 4), a history of acute coronary syndrome or cardiovascular disease, a body mass index of 30 kg/m^2^ or higher, systolic blood pressure outside the range of 115–160 mmHg, not within 75–100 mmHg for diastolic, antihypertensive use, or intake of dietary supplements such as protein and creatine.

We calculated the number of participants required using the G*Power 3.1.9.2 (Kiel, Germany) program, targeting a statistical power of 0.80 and a significance level of 0.05. The effect size was set to 0.22, corresponding to a Cohen’s *d* of 0.44, based on the findings of Seo et al. [15] for midthigh muscle total muscle volume changes in older adult women with sarcopenia after 16 weeks of resistance training. Cohen’s *d* was converted to an *F*-effect size suitable for the analysis of variance. Considering a potential dropout rate of 20%, 36 participants were recruited to ensure sufficient statistical power and detect statistically significant differences between groups.

All participants were informed of the study’s purpose, methods, and risks and provided written informed consent.

### 2.3. Intervention

#### 2.3.1. Resistance Exercise Protocol

RE is widely recognized as an evidence-based rehabilitation method for enhancing muscle strength and physical function, particularly after cartilage regenerative surgery. In this study, we implemented a standardized RE program as a key part of the intervention for both the NG and PG groups [16]. The program started three days post-surgery and consisted of two phases: a non-weight-bearing (NWB) phase for the first six weeks and a full-weight-bearing (FWB) phase for the next six weeks. Participants attended 24 supervised sessions over 12 weeks, with sessions held twice a week on consistent days (Mondays and Thursdays).

The RE program included three core components: warm-up, strengthening exercises, and cool-down activities. In the NWB phase, participants performed resistance exercises using Thera-Bands with progressive resistance levels, starting with light resistance (yellow) and progressing to moderate resistance (red) based on perceived exertion. Strengthening exercises targeted key muscle groups, including quadriceps and hamstrings, with gradual adjustments to intensity, repetition, and load.

In the FWB phase, participants transitioned to machine-based resistance exercises, which included leg presses, squats, lunges, and hamstring curls. The progression of repetitions and load was carefully managed to accommodate increasing weight-bearing capacity. Additional balance and proprioceptive exercises were also introduced during this phase to enhance functional stability.

Warm-up and cool-down activities were standardized across both phases to ensure consistency. The complete exercise protocol, including specific exercises, intensity levels, cadence, and repetitions, is detailed in Table 1.

#### 2.3.2. Nitrate Supplementation Details

NO_3_^−^ supplementation can improve muscle function and recovery [17]; however, its effects in postoperative rehabilitation are not completely understood. In this study, we used beetroot juice as a standardized source of NO_3_^−^ to ensure consistent dosing, in addition to evaluating the impact of NO_3_^−^ supplementation when combined with RE.

Both groups received 70 mL beetroot juice, which contained a high concentration of NO_3_^−^ (6.5 mmol NO_3_^−^, Beet It Sport; James White Drinks, Ipswich, United Kingdom) or 70 mL placebo with negligible NO_3_^−^ (0.04 mmol NO_3_^−^, James White Drinks, Ipswich, United Kingdom). The intervention and placebo were administered by a therapist who was not involved in the study. Both drinks contained 18 g of carbohydrates, of which 17 g were sugars, along with 3.7 g of protein and 0.48 g of salt.

Furthermore, the provided placebo had the same packaging, color, smell, and taste, such that neither the participants nor the researchers were aware of which drink they were consuming. Additionally, both drinks were consumed 2 h before participants were included in each RE intervention session, where they were instructed to avoid eating foods rich in NO_3_^−^, such as beets, celery, and spinach, for 48 h before the NO_3_^−^ supplementation. Notably, peak plasma concentrations of nitrate after oral administration were not measured in this study because the primary focus was on evaluating the clinical and functional outcomes of NO_3_^−^ supplementation combined with resistance exercise in postoperative rehabilitation. Therefore, future research may incorporate pharmacokinetic analyses to better understand the absorption and bioavailability of nitrate and its potential contributions to observed effects.

### 2.4. Primary Outcome

#### 2.4.1. Thigh Muscle Cross-Sectional Area

The primary outcome of this study was the assessment of muscle mass by measuring thigh muscle cross-sectional area (CSA) using magnetic resonance imaging (Sigma TwinSpeed, GE Healthcare, Chicago, IN, USA). Measurements were performed in the supine position with the knee extended in the MRI machine using Velcro straps to restrict knee movement. Furthermore, T2-weighted axial images were obtained at 8 mm intervals, with an echo time of 119 ms, a repetition time of 6120 ms, and a 400 mm field of view on a 512 × 512 matrix. The thigh muscle CSA was measured by initially confirming the length from the greater trochanter to the lateral epicondyle using full-length femur radiography and subsequently measuring a T2-enhanced cross-section at 50% of this length. The measurements were calculated using the accessible regions of interest (ROI) tool of the Picture Archiving and Communication System program (Viewrex, TECHHEIM, Guro, Republic of Korea), outlined along the fascia and calculated values, and performed twice by a radiologist with an interval of 72 h between the measurements [18] (Supplementary Figure S1). The reliability of the measured values was evaluated using Cronbach’s alpha coefficient, and values >0.8 were used in the analysis. Thigh muscle CSA was assessed at baseline and 12 weeks after the intervention.

#### 2.4.2. Knee Strength

The knee strength was another primary outcome evaluated. We used isokinetic dynamometry (HUMAC Norm; CSMi, Stoughton, MA, USA) to measure maximal voluntary isometric contraction (MVIC) during extension and flexion. The participant was initially asked to sit on a chair with the hip and knee joints flexed at 90° and 60°, respectively. Next, the trunk and pelvis were secured to the chair using a seatbelt. Additionally, the leg was secured with an ankle strap 3 cm above the lateral malleolus of the fibula, and the axis of rotation of the dynamometer was made to align with the lateral epicondyle of the femur. Subsequently, participants performed submaximal contractions twice in extension and flexion with a 5 s rest period between sets. Verbal feedback and encouragement were provided to maximize contraction, and a monitor was used to display torque values during the test. After a 10 s rest period following the submaximal contraction, two 5 s MVICs of the knee extensor were measured, with a 5 s rest period between contractions. After measuring the knee extensors, the MVIC of the knee flexors was measured in the same manner. We used the highest of both measurements in the analysis, which was expressed as a percentage (%) of peak torque/body weight [19]. In addition, knee strength was assessed at baseline and at 6 and 12 weeks after the intervention.

### 2.5. Secondary Outcome

#### 2.5.1. Sarcopenia-Related Outcomes

We assessed sarcopenia-related outcomes using two key measures—the Short Physical Performance Battery (SPPB) and skeletal muscle index (SMI). Physical performance and muscle mass were comprehensively evaluated using these measures, providing a robust assessment of sarcopenia.

The SPPB is useful for measuring lower extremity function and is an objective tool that can predict impairment in mobility during activities of daily living [20]. This test tool comprises balance, gait speed, and repeated chair stand test items. It has a score that ranges from 0 (lowest performance) to 4 (highest performance), depending on the performance of each item, with 12 points in total.

Furthermore, measurements were performed in the following order for the balance test: side-by-side, semi-tandem, and tandem. The normal and semi-tandem stances were scored 1 point each for 10 s, whereas the tandem stance was worth 1 point for ≥3 s and 2 points for ≥10 s. Failure to maintain the normal position for 10 s resulted in a score of 0, which indicated failure to perform.

For the gait speed test, 0 points were allocated for not being able to walk. However, 1 point was allocated for >8.7 s, 2 points for 6.21–8.7 s, 3 points for 4.82–6.20 s, and 4 points for <4.82 s of walking. The test was performed twice, and the participants were allowed to use crutches or walking aids thereafter.

Finally, we used the repeated chair stand test to measure the time to sit down and stand up from a chair with the arms crossed over the chest five times. We assigned a score of 0 for >60 s, 1 for 16.7–60 s, 2 for 13.7–16.7 s, 3 for 11.2–13.7 s, and 4 for <11.2 s. However, no verbal encouragement was provided during the test to ensure that the results reflected the participants’ natural performances. We stopped the test if the use of hands or arms or a fall was anticipated, which resulted in a score of zero. After completing the test, the measured values were summed into composite scores and used for the analysis. Notably, measurements were taken at baseline and 12 weeks after the intervention. The test-retest reliability of the SPPB instrument was 0.87, and convergent validity was *p* = 0.015 [20].

Additionally, we evaluated SMI using a body water analyzer (BWA 2.0; InBody Co., Gangnam, Korea) following the principle of bioelectrical impedance analysis. According to the manufacturer’s guidelines, measurements were performed in the morning after the participants fasted for at least 2 h, and all objects that could conduct an electric current were removed from the body. The room temperature was maintained at 20–25 °C during the measurement, and the participants were in the supine position on the bed for at least 10 min to reduce the movement of body fluids. Measurements were performed with clamp-like electrodes placed on both the wrists and ankles, ensuring no contact between the participant’s arms and torso, arms approximately 15° apart, no contact between thighs, and legs approximately shoulder-width apart. SMI measurements were performed at baseline and at 6 and 12 weeks after the intervention.

#### 2.5.2. Osteoarthritis-Related Outcome

We measured osteoarthritis-related outcomes to evaluate knee joint function, symptoms, and physical limitations associated with osteoarthritis. Two validated tools, the International Knee Documentation Committee (IKDC) questionnaire and the Western Ontario and McMaster Universities Osteoarthritis Index (WOMAC), were used to provide a comprehensive analysis of knee health and patient-reported outcomes.

The IKDC questionnaire is used to assess symptoms of knee OA and other knee-related injuries. This tool is used to evaluate the overall health of the knee joint, including symptoms, limitations in daily activities, and restrictions in sports because of damage caused by conditions such as ligament injury or OA. The questionnaire is divided into three categories: symptoms, sports activities, and knee function, with 18 questions in total. Responses are converted to a normalized score ranging from 0–100, with 100 indicating no symptoms or limitations. In this study, we divided the summed raw scores by the maximum possible score (87) and multiplied by 100 to standardize the results. The internal consistency of this screening tool was 0.91, and construct validity was *p* < 0.01 [21].

In addition, the WOMAC is a standardized questionnaire that is used to assess the condition of patients with knee OA, including pain, stiffness, and joint function. Higher scores indicate greater pain, stiffness, and functional limitations. This questionnaire includes 5 items for pain (score range: 0–20), 2 for stiffness (score range: 0–8), and 17 for functional limitations (score range: 0–68), with 96 points in total. The test-retest reliability for pain, stiffness, and function (activities of daily living) was 0.91, 0.89, and 0.90, respectively, and the construct validity was *p* < 0.01 [22].

### 2.6. Statistical Analysis

The statistical program Statistical Package for Social Sciences (SPSS) for Windows (Version 23.0, IBM Corp., Armonk, NY, USA) was used to analyze the data. Data distribution was determined using the Shapiro–Wilk test. For the homogeneity test, the independent *t*-test was used for normally distributed dependent variables among the continuous data, and the Mann–Whitney *U* test was used for MVIC of the knee extensor and SPPB that were not normally distributed. Additionally, Fisher’s exact test was used to test the homogeneity of the nominal data. Two-way repeated-measures measures analysis of variance was used to test for differences in normally distributed dependent variables. Mauchly’s test for sphericity was applied, with sphericity assumed to be *p* > 0.05, and Wilks lambda was used if *p* < 0.05. Post hoc analyses were performed with a simple main effect using the Bonferroni correction. Furthermore, generalized estimating equations (GEEs) were used to analyze variables that did not follow a normal distribution, such as the MVIC of the knee extensor and SPPB, which were used to evaluate interaction effects (e.g., time × group) in repeated measures. In addition, within- and between-group differences were determined using the Wilcoxon signed-rank test and the Mann–Whitney *U* test with Bonferroni correction, respectively. Effect sizes for repeated measures analysis and GEEs were calculated using partial eta squared ($\eta_{p\;}^{2}$), and the resulting values were interpreted as follows: small effect (0.01 ≤ $\eta_{p\;}^{2}$ < 0.06), medium effect (0.06 ≤ $\eta_{p\;}^{2}$ < 0.14), and large effect ($\eta_{p\;}^{2}$≥ 0.14). The last observation carried forward (LOCF) method was used to handle missing data due to participant dropout. This was done to ensure adherence to the intent-to-treat (ITT) principle. The LOCF method replaced missing values with the last observed value for each variable. All statistical significance was defined as *α* < 0.05.

## 3. Results

We included 36 participants in the study; however, two participants from the NG dropped out (one at 6 weeks and the other at 12 weeks). Similarly, two participants from the PG dropped out at 6 weeks, requesting discontinuation of the intervention. Hence, we had a final sample size of 16 participants in each group.

Additionally, we collected preintervention data at 8.92 ± 2.71 d before the intervention and postintervention data at 3.91 ± 2.46 d after the intervention. Similar compliance with the intervention was observed in both groups, with attendance rates of 94.79 ± 5.38% in the NG group and 94.53 ± 5.43% in the PG group (*p* = 0.892). These results confirmed equal participation between groups, supporting the internal validity of the study.

Baseline characteristics were comparable between the two groups, with no statistically significant differences observed (Table 2).

### 3.1. Primary Outcomes

#### 3.1.1. Thigh Muscle Cross-Sectional Area

The thigh muscle CSA showed a statistically significant interaction effect of time × group (*p* = 0.049, $\eta_{p\;}^{2}$= 0.11) and a statistically significant difference between time points (*p* < 0.001). Post hoc analysis revealed no significant change in the CSA for the NG. However, it reduced statistically significantly in the PG at 12 weeks after the intervention (*p* < 0.001).

#### 3.1.2. Knee Strength

The extension MVIC showed a statistically significant interaction effect of time × group (*p* = 0.028, $\eta_{p\;}^{2}$= 0.08) and a statistically significant difference between time points (*p* < 0.001). The post hoc analysis indicated that the NG had significantly higher MVIC values than the PG at both 6 weeks (*p* = 0.002) and 12 weeks (*p* = 0.005). However, both groups showed significant increases from baseline to 12 weeks.

Furthermore, the flexion MVIC showed a statistically significant difference between time points (*p* < 0.001); however, no significant interaction of effect of time × group was found (*p* = 0.055, $\eta_{p}^{2}$ = 0.08). The post hoc analysis revealed that the NG significantly improved at 6 weeks (*p* < 0.001) and 12 weeks (*p* < 0.001). Conversely, the PG showed significant improvement only at 12 weeks (*p* = 0.004). Detailed information on the primary outcomes is presented in Table 3.

### 3.2. Secondary Outcomes

#### 3.2.1. Sarcopenia-Related Outcome

The balance test of the SPPB showed a statistically significant interaction effect of time × group (*p* = 0.004, $\eta_{p}^{2}$ = 0.22); however, the time points did not significantly differ (*p* = 0.130). The post hoc analysis revealed no significant changes in the NG (*p* = 0.280, ∆: 0.22 [95% confidence interval (CI): −0.19 to 0.63]), whereas the PG showed a statistically significant decrease at 12 weeks after the intervention (*p* = 0.002, ∆: −0.67 [95% CI: −1.08 to −0.26]).

Notably, the gait speed test did not show a statistically significant interaction effect of time × group (*p* = 0.677, $\eta_{p}^{2}$ = 0.01), and the time points also did not significantly differ (*p* = 0.407).

For the repeated chair stand test, a statistically significant interaction effect of time × group was observed (*p* = 0.040, $\eta_{p}^{2}$ = 0.119); however, the time points did not significantly differ (*p* = 0.337). The post hoc analysis revealed no significant changes in the NG (*p* = 0.415, ∆: 0.17 [95% CI: −0.24 to 0.58]); however, in the PG, there was a statistically significant decrease at 12 weeks after the intervention (*p* = 0.035, ∆: −0.44 [95% CI: −0.85 to −0.03]).

**Figure 3:**
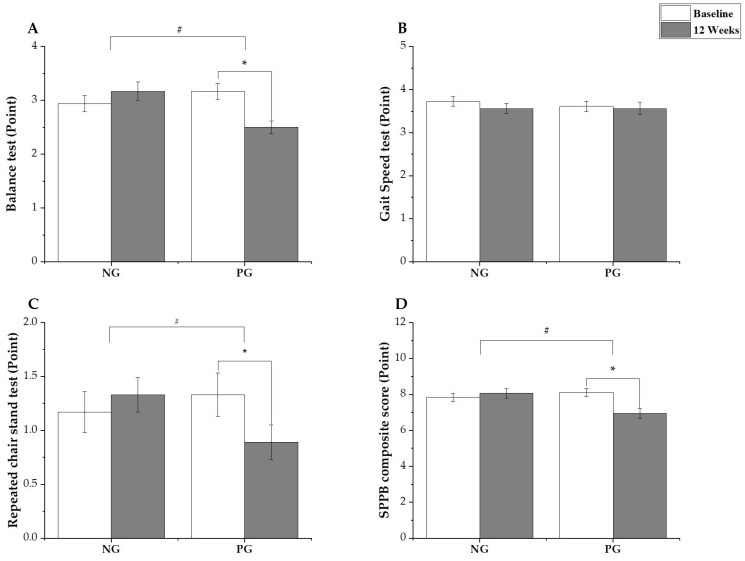
Result of Short Physical Performance Battery (SPPB): (**A**) balance test, (**B**) gait speed test, (**C**) repeated chair stand test, and (**D**) SPPB composite score. Values are expressed as mean and standard error. * Significant differences within the group; ^#^ Interaction effect of time × group by generalized estimation equation.

Additionally, the SPPB composite score showed a statistically significant interaction effect of time × group (*p* = 0.005, $\eta_{p}^{2}$ = 0.21), and the time points showed a statistically significant difference (*p* = 0.049). The post hoc analysis revealed no significant changes in the NG (*p* = 0.502, ∆: 0.22 [95% CI: −0.44 to 0.89]), whereas, in the PG, a statistically significant decrease was noted at 12 weeks after the intervention (*p* = 0.001, ∆: 1.17 [95% CI: −1.83 to −0.50]). The SPPB results are shown in Figure 3A–D.

Additionally, the SMI did not show a significant interaction effect of time × group (*p* = 0.253, $\eta_{p}^{2}$ = 0.04), and the time points did not significantly differ (*p* = 0.194).

#### 3.2.2. Osteoarthritis-Related Outcome

The IKDC score showed a statistically significant interaction effect of time × group (*p* = 0.030, $\eta_{p}^{2}$ = 0.2), and the time points also statistically significantly differed (*p* < 0.001). The post hoc analysis revealed that in the NG, there were significant improvements at 6 weeks (*p* = 0.047, ∆: 2.24 [95% CI: −0.07 to 4.54]) and 12 weeks (*p* < 0.001, ∆: 8.03 [95% CI: 5.67 to 10.38]) after the intervention. However, in the PG, significant improvement was observed only at 12 weeks after the intervention (*p* = 0.001, ∆: 4.04 [95% CI: 1.69 to 6.39]).

**Figure 4:**
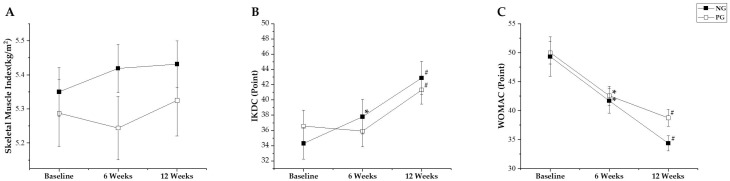
Result of secondary outcome: (**A**) skeletal muscle index (SMI), (**B**) International Knee Documentation Committee (IKDC), and (**C**) Western Ontario and McMaster Universities Osteoarthritis Index (WOMAC). Values are expressed as mean and standard error. * significant differences from baseline to 6 weeks; ^#^ significant differences from baseline to 12 weeks.

The WOMAC score showed a statistically significant difference within the groups (*p* < 0.001); however, there was no significant interaction effect of time × group (*p* = 0.244, $\eta_{p}^{2}$ = 0.04). The post hoc analysis revealed that both the NG and PG decreased at 6 weeks (*p* < 0.001, ∆: −7.43 [95% CI: −11.07 to −3.78]; *p* = 0.001, ∆: −6.61 [95% CI: −10.26 to −2.97], respectively) and 12 weeks (*p* < 0.001, ∆: −13.93 [95% CI: −18.24 to −9.62]; *p* < 0.001, ∆: −10.00 [95% CI: −14.31 to −5.69], respectively) after the intervention. The SMI, IKDC, and WOMAC results are shown in Figure 4A–C.

## 4. Discussion

In this study, we investigated the combined effects of RE and NO_3_^−^ supplementation in middle-aged women with sarcopenia undergoing MSC implantation. The findings revealed that NO_3_^−^ supplementation, when combined with RE, effectively preserved muscle mass and significantly improved knee extension strength. Hence, this intervention can potentially improve postoperative rehabilitation outcomes.

MSC implantation is safe and effective for managing OA. It reduces pain and inflammation and enhances mobility [23]; however, challenges such as postoperative muscle atrophy resulting from its use have not been solved. Notably, thigh muscle atrophy increases the risk of complications, such as falls and balance impairments [24]. These issues are exacerbated in patients with sarcopenia because their low SMI negatively impacts rehabilitation outcomes. Liao et al. [5] reported that patients with sarcopenia experience less improvement in mobility and physical function post-surgery compared with nonsarcopenic patients. Therefore, strategies to prevent rapid thigh muscle atrophy are essential to restore mobility and physical function in this population.

In this study, patients with sarcopenia, as defined by the Asian Working Group for Sarcopenia criteria, underwent RE combined with 400 mg of NO_3_^−^ supplementation to mitigate postoperative thigh muscle atrophy. The results showed that thigh CSA in the PG significantly reduced, whereas the NG maintained preoperative CSA levels. The prevention of thigh muscle atrophy in the NG could result from the combined effects of enhanced muscle activation and increased strength due to RE and NO_3_^−^ supplementation.

Furthermore, postintervention thigh extension strength increased in both groups. However, it was significantly higher in the NG at 6 and 12 weeks. Similarly, the flexor muscle strength improved more rapidly in the NG than in the PG, highlighting the enhanced effectiveness of the intervention in improving overall muscle performance. The strength-enhancing effects of NO_3_^−^ supplementation in this study are consistent with the findings of Coggan et al. [25] and Sim et al. [26].

In addition, the mechanisms behind the increased strength from NO_3_^−^ supplementation are not fully understood; however, previous studies provide valuable findings. Ferguson et al. [27] found that NO_3_^−^ enhances oxygen delivery to type II muscle fibers, which improves metabolic regulation. Additionally, Hernández et al. [28] reported that NO_3_^−^ supplementation elevates plasma NO_3_^−^ and nitrite levels and upregulates Ca^2+^-regulating receptors such as calsequestrin-1 and dihydropyridine, which enhance contractile function in type II fibers. Furthermore, Campos et al. [29] highlighted the NO_3_^−^-induced reduction of ATP utilization costs, which improves mitochondrial efficiency and increases blood flow to type II fibers; hence, this collectively improves contractility and delays fatigue. These combined effects optimize oxygen and nutrient delivery during resistance exercise, enabling higher workloads and promoting protein synthesis in the muscles [30]. Moreover, mechanical tension in the muscles is increased by NO_3_^−^ supplementation. This tension is important for muscle hypertrophy, with muscle atrophy resulting from insufficient mechanical tension. However, increased tension stimulates muscle growth [31]. Consequently, the prevention of muscle atrophy observed in the NG was likely attributable to heightened muscle stimulation and improvement in strength, which surpassed those observed in the PG.

In addition to the above mechanisms, NO can help prevent muscle damage, support regeneration, and reduce oxidative stress, all of which are crucial for muscle recovery [12,32,33]. Córdova-Martínez et al. [14] reported that NO_3_^−^ supplementation combined with physical activity increased serum creatinine and decreased total protein levels, potentially protecting against sarcopenia by mitigating muscle loss and functional decline [34]. In this study, RE combined with NO_3_^−^ supplementation likely prevented muscle atrophy and improved strength primarily due to improved muscle activation rather than hypertrophy, which is consistent with findings from Esen et al. [35], who observed increased activation in the vastus lateralis following NO_3_^−^ supplementation. Therefore, future studies should further investigate these mechanisms using biomarker analysis.

We also assessed sarcopenia-related factors as secondary outcomes. OA is commonly associated with reduced skeletal muscle mass, a known independent risk factor for sarcopenia [36]. For SPPB, no significant changes were noted in the composite scores from baseline to 12 weeks in the NG, whereas in the PG, a decline of 1.17 points was observed. This clinically meaningful reduction exceeds the established minimal clinically important difference (MCID) of 0.5–1.0 points [37,38], likely reflecting a decline in physical function in the PG. Additionally, the decline in SPPB scores in the PG may be partially attributed to diminished knee extensor strength at earlier stages of the intervention, which could have negatively impacted functional components, such as balance and repeated chair stand performance. However, after 12 weeks, the knee extensor strength in the PG improved from baseline levels, potentially mitigating further functional decline. This recovery in knee strength may be an indirect result of the RE program; however, the improvement was not rapid and consistent, as observed in the NG. Notably, the enhanced knee strength in the NG throughout the intervention period, which was facilitated by NO_3_^−^ supplementation, may have contributed to the stabilizing their SPPB scores and ability to maintain functional independence [39,40]. Hence, future studies should explore whether longer interventions or higher doses of NO_3_^−^ could result in measurable functional improvements over time.

The SMI remained unchanged in both groups, possibly due to the beneficial effects of RE on muscle mass and function and consistent with findings by Beaudart et al. [41]. However, NO_3_^−^ supplementation did not significantly enhance SMI, possibly due to the relatively short intervention period, which is insufficient to cause significant muscle mass changes, as muscle hypertrophy generally requires longer-term interventions. Additionally, the absence of controlled dietary protein intake in this study may have limited the potential for SMI improvement because sufficient protein and essential amino acid intake are crucial for muscle synthesis. In contrast, Liao et al. [5] reported that combining RE with intake of leucine-enriched protein improved SMI in older patients with OA. These findings highlight that proteins and essential amino acid intake significantly promote muscle mass development, aligning with the conclusions of McKendry et al. [42]. Based on these findings, future research should explore the combined effects of NO_3_^−^ supplementation and protein intake on SMI to determine whether their synergistic interaction can enhance muscle mass.

Regarding OA symptoms analyzed as secondary outcomes, the IKDC scores in the NG improved significantly at both 6 and 12 weeks postintervention, whereas in the PG, significant improvements were observed only at 12 weeks. These findings suggest that early improvements in knee strength, facilitated by NO_3_^−^ supplementation, may have contributed to the enhanced IKDC scores observed in the NG. Moreover, knee strength is a key predictor of performance in functional mobility tests, such as the modified physical performance test and the 6 min walk test [43]. Additionally, strength in knee extensors correlated with stair-climbing ability [44]. The observed changes in IKDC scores in the NG at 12 weeks reached 8.03 points, which was close to but did not fully meet the MCID of 9.8 points for knee function improvement in knee OA populations [45]. This limited extent of IKDC score improvements may stem from the shorter intervention period in this study. Hence, longer-term interventions might be required to achieve clinically meaningful thresholds. Nevertheless, the significant early improvements observed in the NG suggest that NO_3_^−^ supplementation, combined with RE, enhances functional recovery during the postoperative period. In contrast, the PG showed a 4.04-point improvement at 12 weeks, which falls short of the MCID threshold even though the improvement was significant, reflecting a more gradual recovery trajectory.

Improvements in the WOMAC score were observed in both groups; however, the difference was not significant. This suggests that, in the short term, OA symptoms like pain are more likely alleviated by surgical intervention rather than changes in muscle mass or strength. Song et al. [23] reported significant improvements in both IKDC and WOMAC scores 2 years after MSC implantation, with ICRS grade IV cartilage defects improving to grades I and II. These findings highlight that surgical methods significantly contribute to addressing postoperative pain and related symptoms. In this study, the WOMAC scores decreased by 13.93 points in the NG at 12 weeks, falling slightly short of the MCID threshold of 16.1 points [46], whereas in the PG, a smaller decrease of 10.00 points was observed, which also did not meet the MCID. However, notable symptom relief was observed in both groups. The results indicate that a longer intervention period or additional therapeutic components may be necessary to achieve clinically meaningful improvements in WOMAC scores, as defined by the MCID.

The present study provides new insights into the role of NO_3_^−^ supplementation combined with RE in improving postoperative outcomes among middle-aged women with sarcopenia undergoing MSC implantation for OA. Our findings suggest that this combined intervention effectively prevents muscle atrophy and enhances knee extensor strength, which is critical for rehabilitation. Previous research highlighted the benefits of NO_3_^−^ supplementation in muscle performance and recovery; however, to the best of our knowledge, this is the first clinical trial to examine its combined impact with RE in a postoperative setting. The results are particularly significant given the high prevalence of sarcopenia and its negative effect on post-surgical recovery, as described in earlier studies on orthopedic outcomes and muscle degeneration. Our study findings reveal the potential of NO_3_^−^ supplementation to complement established rehabilitation strategies and suggest that targeting muscle strength early in the recovery process may accelerate functional improvements. Clinicians can potentially prevent muscle atrophy and enhance recovery by integrating NO_3_^−^ supplementation (400 mg, administered 2 h prior to exercise) and RE (2–3 sessions per week over 12 weeks) into postoperative protocols. Furthermore, these interventions could have broader use in community rehabilitation settings, supporting accessibility and scalability for diverse patient populations.

This study has some limitations. First, the combined effects of RE and NO_3_^−^ supplementation were assessed, making it difficult to isolate the impact of NO_3_^−^ alone. Second, the intervention period was relatively short. We evaluated the thigh muscle CSA as the primary outcome and found that RE combined with NO_3_^−^ supplementation prevented thigh muscle atrophy. However, we were unable to confirm muscle hypertrophy in the NG, and we believe that a study with a longer intervention period is needed to confirm this. Third, the exclusion of patients with Kellgren–Lawrence grade IV OA limits the generalizability of the findings, as the study results may not be applicable to individuals with more severe osteoarthritis. This exclusion was necessary due to contraindications for MSC implantation in these cases in Korea. Additionally, the study population comprised exclusively middle-aged women, which further limits the applicability of the findings to other demographic groups, such as men, younger individuals, or those without sarcopenia. Moreover, although all participants were postmenopausal, we did not collect detailed data on the number of years since menopause. Given that hormonal changes associated with menopause affect muscle mass and function, variability in postmenopausal duration may have influenced the observed outcomes. Future studies should consider including this variable to better understand its potential impact on muscle adaptations. Fourth, we did not control for or monitor the participants’ daily dietary habits, which could influence muscle function and recovery. The participants were instructed to avoid foods rich in NO_3_^−^; however, other aspects of their diet were not closely regulated. Hence, future studies should consider dietary monitoring to minimize variability. Fifth, we did not include blood biomarker analysis, which could have provided valuable insights into the underlying mechanisms of the observed effects. The intervention caused improvements in muscle strength and CSA; however, blood biomarkers such as inflammatory cytokines, muscle damage markers, or metabolic indicators could have enhanced the understanding of the physiological pathways influenced by NO_3_^−^ supplementation and resistance exercise. Finally, we used a relatively small sample size, which may limit the generalizability of the findings. The initial sample size was calculated to achieve adequate statistical power; however, the dropout of four participants could have further reduced the study’s statistical power. To address this, we applied the LOCF method to include all participants in the analysis, adhering to the intent-to-treat principle. However, future research with larger cohorts is necessary to confirm these findings and enhance their applicability to broader populations.

## 5. Conclusions

Our findings reveal that NO_3_^−^ supplementation, when combined with RE, effectively prevented thigh muscle atrophy and enhanced postoperative muscle strength. Furthermore, the improvements in IKDC scores in the NG suggest that NO_3_^−^ supplementation may accelerate early functional recovery, particularly through enhanced knee strength. Moreover, the combined intervention of NO_3_^−^ supplementation and RE could mitigate sarcopenia-related symptoms and atrophy and alleviate OA-related impairments, including reduced joint function and mobility. However, while the intervention effectively maintained muscle mass, as indicated by SMI, it did not cause significant muscle hypertrophy within the short intervention period. The clinical significance of secondary outcomes, such as SPPB scores, should also be interpreted with caution due to their limited implications within the short intervention period. This approach is a viable strategy for preventing postoperative muscle atrophy and improving muscle strength in middle-aged women with sarcopenia, potentially enhancing rehabilitation outcomes and quality of life. However, future research should explore long-term interventions and include patients with various stages of OA to further validate and expand on these findings. These results provide a basis for integrating NO_3_^−^ supplementation and RE into rehabilitation protocols in sports medicine, providing a novel approach to patient recovery after surgery.

## Figures and Tables

**Table 1:** Resistance exercise intervention programs.

Phase	Classification	Exercise	Cadence	Intensity (OMNI Scale)	Repetition
NWB Phase (0–6 weeks)	Warm-up	UBC	-	-	5 min
		Stretching	-	-	10 min
	Strengthening	Q/H setting	-	-	10 s × 10 reps
		Four-way SLR	-	-	10 s × 10 reps
		Knee extension with Thera-band (Yellow/red)	Moderate	4–6	12 reps × 5 sets (by 3 weeks) 8 reps × 4 sets (by 4–6 weeks)
		Hamstring curl with Thera-band (Yellow/red)	Moderate	4–6	
	Additional exercise	Ankle dorsi/plantar flexion with Thera-band (Yellow/red)	Moderate	4–6	12 reps × 5 sets
		Hip ab/adduction			
	Cool-down	Cool-down			5 min
FWB Phase (6–12 weeks)	Warm-up	Stationary bike	-	-	5 min
		Stretching	-	-	10 min
	Strengthening	Leg extension with machine	Moderate to Slow	4–6	12 reps × 5 sets (by 9 weeks) 8 reps × 4 sets (by 9–12 weeks)
		Hamstring curl with machine	Moderate to Slow	4–6	
		Leg press	Moderate to Slow	4–6	
		Squat	Moderate to Slow	4–6	
		Lunge	Moderate to Slow	4–6	
	Additional exercise	Balance and proprioceptive exercises	-	-	10 min
	Cool-down	Cool-down			5 min

UBC: upper body cycle; ROM: range of motion; Q/H: quadriceps/hamstring; SLR: straight leg raises; NWB: non-weight-bearing; FWB: full-weight-bearing.

**Table 2:** Baseline characteristics (*N* = 36).

	NG (*n* = 18)	PG (*n* = 18)	Δ (95% CI)	*p* Value
Age (years)	59.44 ± 3.24	59.33 ± 4.00	0.11 (−2.35 to 2.58)	0.928 ^†^
Height (cm)	157.93 ± 4.04	156.71 ± 4.27	1.22 (−1.64 to 4.08)	0.391 ^†^
Weight (kg)	59.57 ± 5.18	60.02 ± 4.97	−0.45 (−3.95 to 3.04)	0.795 ^†^
BMI (kg/m^2^)	23.92 ± 2.32	24.45 ± 1.95	−0.53 (−2.02 to 0.95)	0.468 ^†^
KL grade 2 (*n*)	4 (22.2%)	5 (27.8%)		0.700 ^§^
KL grade 3 (*n*)	14 (77.8%)	13 (72.2%)		
Right knee (*n*)	9 (50.0%)	8 (44.4%)		0.738 ^§^
Left knee (*n*)	9 (50.0%)	10 (55.6%)		
CSA (cm^2^)	76.96 ± 5.38	75.25 ± 3.90	1.72 (−1.47 to 4.90)	0.281 ^†^
Knee Extension MVIC (%)	71.52 ± 21.62	61.42 ± 18.45	10.09 (−3.52 to 23.71)	0.084 ^‡^
Knee Flexion MVIC (%)	35.17 ± 16.27	31.53 ± 12.02	3.63 (−6.06 to 13.32)	0.451 ^†^
SMI (kg/m^2^)	5.35 ± 0.27	5.29 ± 0.37	0.06 (−0.16 to 0.28)	0.602 ^†^
HGS (kg/f)	14.72 ± 2.00	14.87 ± 1.69	−0.14 (−1.40 to 1.11)	0.816 ^†^
IKDC (point)	34.94 ± 8.40	36.28 ± 7.82	−1.35 (−6.85 to 4.15)	0.621 ^†^
WOMAC (point)	49.31 ± 12.85	50.07 ± 7.55	−0.76 (−7.90 to 6.38)	0.930 ^†^
Balance test (point)	2.94 ± 0.64	3.17 ± 0.62	−0.22 (−0.65 to 0.20)	0.292 ^‡^
Gait Speed test (point)	3.72 ± 0.46	3.61 ± 0.50	0.11 (−0.22 to 0.44)	0.486 ^‡^
Chair Standing test (point)	1.17 ± 0.79	1.33 ± 0.84	−0.17 (−0.72 to 0.38)	0.575 ^‡^
SPPB composite (point)	7.83 ± 0.99	8.11 ± 0.96	−0.28 (−0.94 to 0.38)	0.443 ^‡^

NG: nitrate supplementation group; PG: placebo supplementation group; BMI: body mass index; SBP: systolic blood pressure; DBP: diastolic blood pressure; KL: Kellgren–Lawrence; CSA: cross-sectional area; MVIC: maximal voluntary isometric contraction; SMI: skeletal muscle index; HGS: handgrip strength: IKDC: International Knee Documentation Committee; Womac: Western Ontario and McMaster Universities Osteoarthritis Index. Data are expressed as mean ± SD or number (percentage).^†^ Independent *t*-test. ^‡^ Mann–Whitney *U* test. ^§^ Fisher’s exact test.

**Table 3:** Comparison of the primary outcomes.

	Baseline	6 Weeks	12 Weeks	Mean Change (95% CI) ^†^	Mean Change (95% CI) ^‡^	Mean Difference (95% CI) ^§^	Mean Difference (95% CI) ^††^
Thigh Muscle Cross-Sectional Area (cm^2^)							
NG (*n* = 18)	76.96 ± 5.38	-	75.57 ± 6.5	-	−1.39 (−2.94 to 0.15)	-	3.92 (0.12 to 7.71)
PG (*n* = 18)	75.25 ± 3.90	-	71.65 ± 4.53	-	−3.59 * (−5.14 to −2.05)		
Knee Extension Maximal Voluntary Isometric Contraction (%)							
NG (*n* = 18)	71.52 ± 21.62	74.85 ± 18.15	92.24 ± 19.88	3.31 (−4.17 to 10.8)	22.88 * (15.42 to 30.33)	18.98 * (9.01 to 28.96)	16.76 * (5.46 to 28.07)
PG (*n* = 18)	61.42 ± 18.45	55.87 ± 10.20	75.48 ± 12.72	−6.25 (−1.24 to 13.74)	15.81 * (8.36 to 23.27)		
Knee Flexion Maximal Voluntary Isometric Contraction (%)							
NG (*n* = 18)	35.17 ± 16.27	45.67 ± 16.12	47.00 ± 11.95	10.50 * (5.96 to 15.04)	11.83 * (6.84 to 16.82)	11.24 (1.87 to 20.62)	7.91 (0.38 to 15.44)
PG (*n* = 18)	31.53 ± 12.02	34.42 ± 11.11	39.09 ± 10.22	2.89 (−1.65 to 7.43)	7.56 * (2.56 to 12.55)		

NG: nitrate supplementation group; PG: placebo supplementation group; CI, confidence interval. Data are expressed as mean ± SD. Thigh muscle cross-sectional area and knee flexion maximal voluntary isometric contraction were analyzed using two-way repeated measures analysis of variance, and knee extension maximal voluntary isometric contraction was analyzed using generalized estimation equations. ^†^ Mean change (baseline vs. 6 weeks after the intervention); ^‡^ Mean change (baseline vs. 12 weeks after the intervention); ^§^ Mean difference at 6 weeks after the intervention; ^††^ Mean difference at 12 weeks after the intervention. * Significant difference in post hoc analysis.

## Data Availability

The data that support the findings of this study are available from the corresponding author upon reasonable request.

## Supplementary Materials

The following supporting information can be downloaded at: https://www.mdpi.com/article/10.3390/jcm14020615/s1, Figure S1: Method of thigh muscle cross-sectional area (CSA) measurement.

## Abbreviations

CSA: cross-sectional area; SPPB: Short Physical Performance Battery; IKDC: International Knee Documentation Committee; WOMAC: Western Ontario and McMaster Universities Osteoarthritis Index; MSC: mesenchymal stem cell; NO: nitric oxide; NO_3_^−^: nitrate; OA: osteoarthritis; RE: resistance exercise; ICRS: International Cartilage Repair Society; SMI: skeletal muscle index; MRI: magnetic resonance imaging; ES: effect size; MVIC: maximal voluntary isometric contraction; LOCF: last observation carried forward; ITT: intent-to-treat.
