# Supplementary material for: Impact of Resistance Exercise and Nitrate Supplementation on Muscle Function and Clinical Outcomes After Knee Osteoarthritis Surgery in Middle-Aged Women with Sarcopenia: A Randomized, Double-Blind, Placebo-Controlled Clinical Trial
Source: J Clin Med. 2025 Jan 18;14(2):615. doi: 10.3390/jcm14020615 (PMC11765574; doi:10.3390/jcm14020615)
Supplement: Supplementary file 1 [file jcm-14-00615-s001.zip › jcm-3332341-supplementary.pdf]

Supplementary Material

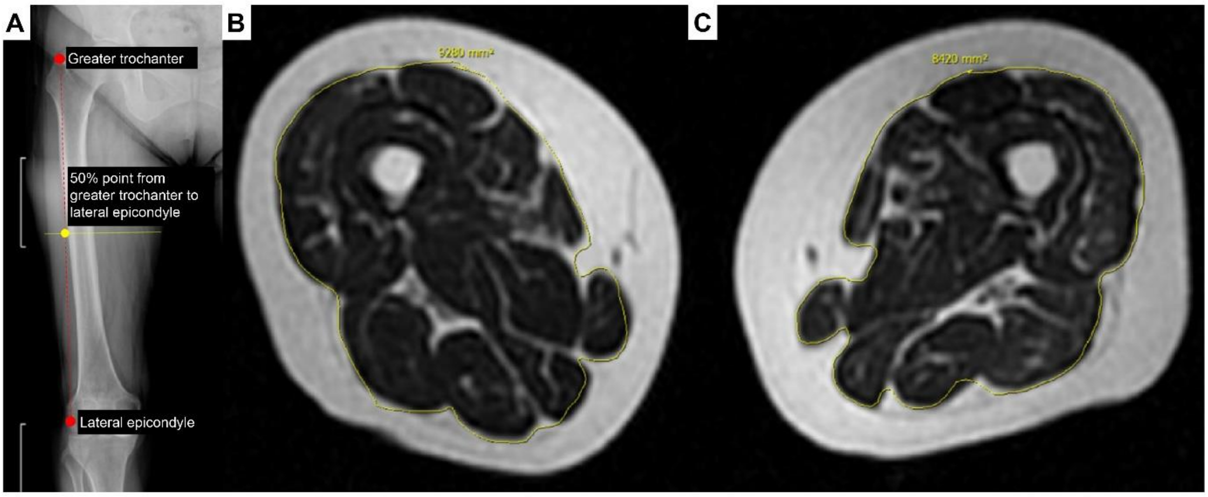

Supplementary Figure S1. Method of thigh muscle cross-sectional area (CSA) measurement: A) The length of the femur was measured using sonography and MRI T2-weighted axial images at the 50% point. B & C) Thigh muscle CSA in a 58-year-old woman: B) Uninvolved side (92.8 cm^2) and C) Involved side (84.2 cm^2)
